# Cytogenetic characterization of *Partamona cupira* (Hymenoptera, Apidae) by fluorochromes

**DOI:** 10.1590/S1415-47572010005000029

**Published:** 2010-06-01

**Authors:** Jefferson de Brito Marthe, Silvia das Graças Pompolo, Lucio Antônio de Oliveira Campos, Tânia Maria Fernandes Salomão, Mara Garcia Tavares

**Affiliations:** Departamento de Biologia Geral, Universidade Federal de Viçosa, Viçosa, MGBrazil

**Keywords:** B chromosome, SCAR, stingless bees

## Abstract

Four colonies of the stingless bee *Partamona cupira* (Hymenoptera: Apidae) were cytogenetically analyzed using conventional staining and the fluorochromes CMA_3_ e DAPI. The females have 2n = 34 chromosomes (2K = 32 $\bar{M}$+2 $\bar{A}$). Some females, however, presented an additional large B acrocentric chromosome, to a total of 2n = 35. Chromosome B and the chromosomal pairs 2, 9 and 10 showed CMA _3_^+^ bands, indicating an excess of CG base-pairs. A clear association was verified between the *P. helleri* B chromosome SCAR marker and the presence of a B chromosome in *P. cupira.* The data obtained suggests that B chromosomes in *P. helleri* and *P. cupira* share a common origin.

Stingless bees of the genus *Partamona* (Hymenoptera, Apidae) are widely distributed geographically. Their range extends from the south of Mexico to south Brazil, spreading northwards along the Pacific coast until Peru (Camargo, 1980).

The cytogenetic characterization of eight species of the genus *Partamona*, viz., *P.pearsoni,P. helleri* (cited as *P.cupira* by Costa *et al.*, 1992), *P. mulata*, *P. ailyae*, *P. vicina*, *P.* sp. aff*. nigrior*, *P. peckolti* and *P. seridoensis* (revision in Brito *et al.*, 2005) showed that all of the females have 2n = 34 chromosomes and that only *P. helleri* presented 0 to 7 B chromosomes.

B chromosomes of *P. helleri* were cytogenetically characterized using C, Q and NOR banding, GTG method, CMA_3,_ DAPI and FISH (Brito *et al.*, 2005). Brito *et al.* (2005) concluded that *P. helleri* B chromosomes are heterochromatic. Genomic DNA treatment with the *Eco*RI restriction enzyme, and Southern blot analysis using an 18S rDNA probe from maize, demonstrated that individuals with B chromosomes displayed bands which were not present in individuals that did not bear this chromosome (RM Brito and SG Pompolo, unpublished data). Thus, the presence of specific sequences in the B chromosomes of this species can be suggested.

Using molecular techniques, Tosta *et al.* (2004) identified one RAPD marker in B chromosome-bearing individuals of *P. helleri*. This RAPD marker was cloned, sequenced and then transformed into a SCAR marker (Tosta *et al.*, 2007). Further on, the presence of this SCAR marker was noted in *P*. *cupira* and *P. criptica* (VC Tosta, personal communication).

Considering that in *P. helleri* the SCAR marker is present exclusively in individuals possessing B chromosomes, and that this marker was also identified in *P. cupira*, the aim of this study was to cytogenetically characterize the latter species, to check for the presence of B chromosomes. As the presence of a B chromosome was detected in some individuals of *P. cupira*, an additional molecular analysis was carried out by using the SCAR marker previously described, in order to check whether there is an association between this sequence and the presence of B chromosomes in this species.

The cytogenetic analyses were carried out with 19, 17, 21 and 11 post-defecating larvae from four *Partamona cupira* colonies (GUI 1, GUI 2, GUI 3 and GUI 11) collected at Guimarânia (18°50'38” S, 46°47'35''W), State of Minas Gerais. Metaphasic chromosomes were obtained from *P. cupira* cerebral ganglia according to Imai *et al.* (1988). The remaining parts of each larva were frozen in an ultra-low temperature freezer at -80 °C, to be subsequently used for DNA analysis. After 24 h, the slides were stained with Giemsa diluted in Sorensen's buffer for 20 min at room temperature.

Sequential staining was performed with the use of the fluorochromes: Distamycin/Chromomycin A_3_ (DA/CMA_3_) and Distamycin/4, 6-diamine-2-phenylindole (DA/DAPI) (Schweizer, 1980).

An average of 10 metaphases per specimen was observed. The best images were selected and captured with a Q Color 30 Olympus camera coupled to an Olympus BX-60 microscope. In order to obtain the metaphase images of slides treated with DA/CMA_3_, the WB (l = 330 to 385 nm) filter was used; for DA/DAPI, the WU filter (l = 450-480 nm) was used.

Chromosomes were classified according to Imai (1991) and the karyotypes were mounted using Corel Photo-Paint from CorelDraw X3 and Adobe Photoshop 7.0 softwares.

For the molecular analyses, the larval DNA was obtained according to Waldschmidt *et al.* (1997) and amplified by using SCAR primers specific for *P. helleri* B chromosomes (Tosta *et al.*, 2007). PCR products were separated by electrophoresis in 1% agarose gels in TBE (90 mM Tris-borate pH 8.0, 10 mM EDTA) buffer, stained with ethidium bromide (0.2 μg/mL) and visualized under UV light with AlphaDigiDoc 1201 software.

A comparison between the presence of SCAR marker and the presence of B chromosome in the studied individuals was carried out after the analyses of the gels.

The cytogenetic analyses revealed that *P. cupira* possesses 2n = 34 chromosomes (Figure 1A). The diploid karyotype is comprised of 5 metacentric, 11 submetacentric and a single pair of acrocentric chromosomes, or 2K = 32 $\bar{M}$+2 $\bar{A}$, according to the nomenclature proposed by Imai (1991), whereby $\bar{M}$ may include metacentric and submetacentric chromosomes, and $\bar{A}$ acrocentric and telocentric ones. *Partamona cupira*, therefore, presented the same chromosome number as other species of the same genus that had already been cytogenetically studied (Costa *et al.*, 1992; Brito *et al.*, 1997, 2003, 2005; Brito-Ribon *et al.*, 1999; Tosta *et al.*, 2004). Nevertheless, an analysis of chromosome morphology demonstrated that the *P. cupira* karyotype is different from that of *P. helleri* and *P.seridoensis* (Brito *et al.*, 2005). *Partamona helleri* and *P.seridoensis* have only metacentric chromosomes (M), whereas the species studied herein have acrocentric (A) and metacentric chromosomes.

The obtained data also revealed that, in addition to the regular chromosomal complement, some individuals of *P. cupira* (10 individuals of the GUI 1 colony and 3 individuals of GUI 11) possessed one B chromosome (Figure 1B). These individuals, therefore, had 2n = 35 chromosomes. This B chromosome was considerably larger when compared to those found in *P. helleri* (Costa *et al.*, 1992; Tosta *et al.*, 2004), and in two other species of stingless bees, *Melipona quinquefasciata* (Pompolo, 1992) and *M. rufiventris* (Lopes *et al.*, 2008).

DA/DAPI staining did not reveal the presence of fluorescent bands in any chromosome. DA/CMA_3_ staining, in turn, revealed bands in the terminal portions of the chromosome pairs 2, 9 and 10, as well as on the short arm of the B chromosome, thus demonstrating the existence of repetitive sequences rich in CG in these regions (Figure 1C). These same chromosomes, plus chromosome pair 15, presented CMA_3_ positive bands in *P. helleri* and *P. seridoensis* (Brito *et al.*, 2005). The difference in the number of chromosomes stained by DA/CMA_3,_ as observed in *P. cupira*, and *P. helleri*/*P. seridoensis*, may be related to a process of chromosome evolution. Nevertheless, confirmation requires further comparative studies. Furthermore, Brito *et al.* (2005), using an *in situ* hybridization assay noted that the chromosome pairs 2, 9, 10 and 15 carried cistrons for ribosomal RNA in *P. helleri* and *P. seridoensis*. An association between CMA_3_ bands and the presence of ribosomal DNA sequence sites in the same chromosomal region, had already been observed in other species of Hymenoptera, such as *Trypoxylon albitarse* (Araújo *et al.*, 2000), *Melipona asilvae* (Rocha *et al.*, 2002) and *Partamona peckolti* (Brito *et al.*, 2003). Thus, it is possible that the CMA_3_ positive regions observed in *P. cupira* may be related to rDNA genes.

Molecular analysis revealed a correspondence of the SCAR marker specific for *P. helleri* B chromosomes, and the presence of B chromosomes in *P. cupira*, since in all B-chromosome-bearing individuals in the colonies GUI 1 and GUI 11, the band corresponding to the SCAR marker specific for *P. helleri* B chromosomes was also observed (Figure 2). Moreover, the presence of this marker was not observed in individuals lacking B chromosomes.

The origins of these B chromosomes and their effects on the bearers, although well discussed (López-León *et al.*, 1994; Gutknecht *et al.*, 1995; MacAllister and Werren, 1997; Camacho *et al.*, 2000; Araujo *et al.*, 2001), are far from clear. However, the sequence of the *P. helleri* SCAR marker and that of *P. cupira*, *P. criptica* and *P. rustica* showed a high degree of similarity (data not shown)*.* This, together with the association of this marker to the presence of the B chromosome in *P. cupira*, as demonstrated herein, implies that *P. cupira* B chromosomes may have the same origin as those of *P. helleri*, and that *P. rustica* and *P. criptica* may also posses B chromosomes, although the latter have not yet been characterized cytogenetically. These analyses may clarify the origin of B chromosomes in the genus *Partamona*.

**Figure 1:**
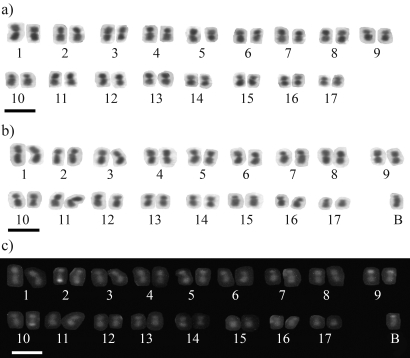
Karyotypes of *Partamona cupira* submitted to Giemsa (A e B: females without and with B chromosomes, respectively) and CMA_3_ (C) staining. Bar = 5 μm.

**Figure 2:**
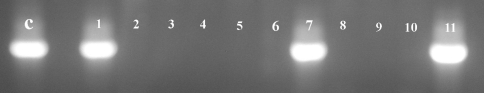
Electrophoretic pattern from genomic DNA of *Partamona cupira* females with (1, 7, 11) and without (2, 3, 4, 5, 6, 8, 9, 10) B chromosome, from GUI 11 colony, amplified with SCARs primers specific for B chromosomes of *P. helleri*. C: a *P. helleri* female from a colony that posses B chromosome, used as control.
